# Efficient Modulation and Processing Method for Closed-Loop Fiber Optic Gyroscope with Piezoelectric Modulator

**DOI:** 10.3390/s19071710

**Published:** 2019-04-10

**Authors:** Michal Skalský, Zdeněk Havránek, Jiří Fialka

**Affiliations:** Central European Institute of Technology, Brno University of Technology, 61200 Brno, Czech Republic; zdenek.havranek@ceitec.vutbr.cz (Z.H.); jiri.fialka@ceitec.vutbr.cz (J.F.)

**Keywords:** fiber-optic gyroscope, Sagnac interferometer, piezoelectric phase modulator, closed-loop configuration, all-fiber, single mode

## Abstract

This paper presents a simple method for compensating the Sagnac phase shift in an interferometric fiber-optic gyroscope (I-FOG) with a piezoelectric modulator. The common advantages of I-FOGs with closed-loop compensation are linearized output characteristics and insensitivity to the light source power, including its time and thermal-induced fluctuations. Whereas closed-loop operation is normally achieved via ramp modulation requiring an electro-optic modulator, all-fiber architectures with a piezoelectric modulator are mostly limited to open loop. Nevertheless, such setups can more conveniently utilize a less expensive single-mode fiber with depolarized light and do not require any custom-made components. The proposed method allows us to combine the advantages of both approaches. Closed-loop compensation is ensured by adding further sinusoidal modulation to the common biasing modulation, such that the Sagnac phase shift is compensated solely at the sampling instants. We describe and experimentally demonstrate the proposed approach, utilizing a test setup to compare our closed-loop solution with open-loop operation. The results denote that the method provides a cost-efficient manner of performance improvement compared to the open-loop I-FOGs based on a piezoelectric modulator.

## 1. Introduction

Using gyroscopes to sense angular motion with respect to the inertial frame is presently essential for many applications, including inertial navigation, attitude and heading reference systems (AHRS), and industry. Together with accelerometers, gyroscopes are part of inertial measurement units providing information about the position in a space without any external reference. These systems are then widely used in aeronautics, submarines, drilling and mining, spacecraft, satellites, and also vehicles such as cars or mobile robots [1,2].

While the less demanding applications usually utilize MEMS (microelectromechanical systems) gyroscopes that exploit the Coriolis force, the requirements of high-grade applications can only be satisfied with optical gyroscopes based on the Sagnac effect. From these, only ring laser gyroscopes (RLGs) and interferometric fiber-optic gyroscopes (I-FOGs) facilitate commercial use. The former have been a leading technology for decades, despite being limited by the lifetime of the He-Ne tube, more difficult construction, and the need of frequency dithering [2,3]. When I-FOGs became commercially available, they were considered a cheaper alternative to RLGs [4]. Currently, the highest-grade I-FOGs using polarization-maintaining (PM) optical fiber and an integrated electro-optic modulator have surpassed the performance of RLGs, achieving a stability better than 0.001${}^{\circ }$/h [5]. In applications such as AHRSs or gyrocompassing, a stability between 0.01–5${}^{\circ }$/h is sufficient, and more attention is paid to cost reduction [3,6]. Thus, multiple solutions replacing PM fiber with ordinary single-mode (SM) fiber were proposed [7,8,9,10,11] and adopted for commercial usage by some of the leading manufacturers [1,12,13]. It was shown in [8] and analyzed in detail by [4] that depolarized (i.e., randomly polarized) light must be used to ensure high stability with SM fiber.

Since the output intensity signal of an I-FOG is given by the interference of two counterpropagating beams with the Sagnac phase shift, its natural response is cosine-shaped. Thus, phase biasing by an electro-optic or a piezoelectric phase modulator (PEM) is necessary to produce the sine-shaped response to allow unambiguous detection and high sensitivity around zero [14]. Whereas the integrated electro-optic modulators used in high-end I-FOGs enable the modulation of only one polarization mode, the PEM is fully compatible with depolarized light; in some low or medium-grade I-FOGs, the PEM may represent the preferred option [10,11,12].

A major advantage of using the PEM consists of no need of specific-purpose optical components; this benefit is associated with the possibility of all-fiber realization of the whole I-FOG, ensuring a simple manufacturing process. The drawback of common solutions utilizing the PEM then lies in their applicability in open-loop operation only; this arises from the fact that ramp modulation, which is used in high-grade closed-loop I-FOGs to compensate the Sagnac effect [13,15], requires a large modulation bandwidth not provided by the PEM. As a result, the output characteristics of I-FOGs equipped with the PEM are usually sine-shaped and their periodicity does not allow unambiguous detection at higher rotation rates.

In the past, there has been proposed several approaches to scale factor linearization using only a piezoelectric modulator [10,16,17,18,19]. The first solutions combined a gating technique [16,17,18,19] with one [16] or more [17] additional slow harmonic modulations, which yielded approximately linear output within a certain range of angular velocities. However, both linearity and bandwidth were limited due to slow compensation during short measurement intervals and analog processing, often requiring a lock-in amplifier. A convenient method of open-loop linearization using lock-in detection and multiple harmonic components ratio was presented in [10]. A problem of sine characteristics linearization occurs when approaching its peak points, decreasing the accuracy and reliability of the method.

In this paper, we propose and explain a novel closed-loop modulation and signal processing method capable of eliminating the major drawbacks of the PEM-based I-FOGs. Contrary to the prior above-mentioned approaches, no lock-in amplifier or complex computing is needed. At the same time, the bandwidth is not limited by low-frequency gating and also the closed-loop phase shift compensation may be ensured within a very wide dynamic rage of measured angular velocity. In the proposed modulation scheme, closed-loop operation is achieved by fully digital signal processing in the field-programmable gate-array (FPGA), allowing a high degree of synchronization between the modulation signal generation and the output intensity signal acquisition.

The following section presents a detailed description and explanation of the proposed closed-loop technique for the PEM in a depolarized I-FOG. Section 3 then discusses the gyro prototype, in which the new method is implemented and tested. The experimental results comparing open and closed-loop operation are outlined in Section 4. Finally, Section 5 summarizes the main conclusions.

## 2. Methods

### 2.1. Phase Modulation in an I-FOG with a PEM

The output intensity signal of an I-FOG is given by the interference of two beams that are equally split into the clockwise (CW) and counterclockwise (CCW) directions. If we consider that Δϕ is the phase shift between the beams, the intensity can be expressed as

(1) $$I=I_{0}\left(1+\cos\Delta \phi \right),$$

where $I_{0}$ stands for the mean light intensity. Assuming the suppression of nonreciprocal effects, such as polarization nonreciprocity and the Faraday or Kerr effects [20], the phase shift Δϕ is equal to the Sagnac phase shift, $\Delta \phi_{s}=K\Omega$, where Ω is the angular velocity and *K* represents the constant of the I-FOG given by its dimensions and used wavelength. Equation (1) indicates that, to achieve sufficient detection sensitivity and unambiguity, the phase shift needs to be biased by ±π/2 to the point with the maximal slope. This is commonly obtained via harmonic or square-wave modulation. After applying synchronous sampling or demodulation [21], the output signal, referred as open-loop, can be expressed as

(2) $$S_{OL}=2I_{0}A\left(\Delta \Phi_{b}\right)\sin\left(\Delta \phi_{s}\right),$$

where $A(\Delta \Phi_{b})$ is a function of the biasing modulation depth, $\Phi_{b}$. The biasing modulation is conveniently realized with the so-called proper frequency, $f_{b}=1/\left(2\tau \right)$, where τ is the transit time of light around the fiber coil. It was shown in [20] that using the proper frequency not only maximizes the phase difference, but also eliminates some major parasitic effects. In addition, the proper frequency enables us to eliminate the zero shift, which may be caused by higher harmonic components in the modulation signal; such an effect is due to the parasitic effects of the PEM, as proposed in [22,23].

**Figure 1:**
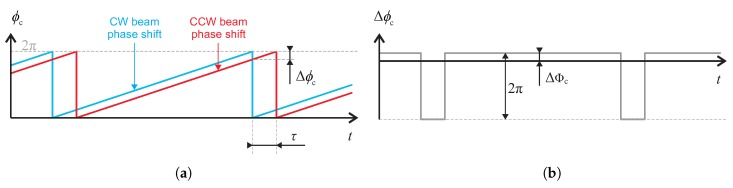
The common compensation with sawtooth modulation: the phase shift introduced to the CW and CCW beams (**a**), and their resulting phase difference (**b**).

As is obvious from Equation (2), the output of open-loop I-FOGs is naturally nonlinear and periodic. This problem is normally solved by additional modulation to compensate the Sagnac phase shift, a solution known as the closed-loop I-FOG [24]. In the closed-loop setup, the ramp modulation scheme (Figure 1) is used to null the Sagnac phase shift induced by the rotation. This technique takes advantage of the time delay τ between the modulation of the beam in the CW and CCW directions as the modulator is placed asymmetrically at one end of the fiber coil. The ramp slope $\Delta \phi_{c}/\tau$ introduces the nonreciprocal shift $\Delta \phi_{c}=-\Delta \phi_{s}$, compensating the Sagnac phase shift. The fundamental frequency of the sawtooth signal is then proportional to the rotation, as firstly proposed by Arditty et al. [25]. The ramp modulation may be performed via either analog serrodyne (as shown in Figure 1a) or digital staircase modulation, where the ramp consists of stairs having a length τ and height $\Delta \phi_{c}$; the effect of the latter on the resulting phase difference (Figure 1b) is identical. The digital phase ramp modulation is also the solution mostly adopted by current closed-loop I-FOGs [1,13]. Due to the steep falling edges (resets) of the ramp modulation, the signal contains a large amount of higher harmonics and requires a wide bandwidth of the modulator. This condition can only be satisfied by electro-optic modulators, fabricated mostly as special-purpose, integrated-optics components. As a result, the all-fiber I-FOGs employing the PEM are limited to open-loop operation, which limits their performance.

A PEM usually consists of a fiber wrapped around a piezoceramic cylinder or a tube in several turns. By applying voltage, the cylinder stretches the fiber and changes the optical path of the light beams [26]. In piezoelectric modulators, a major limiting factor lies in the low modulation rate (up to $10^{4}$–$10^{5}$ Hz) and the presence of mechanical resonances, whose frequencies depend on the dimensions and material of the piezoceramic actuator and can also be influenced by the temperature [27]. The frequency response of a PEM is thus linear within several kHz at most, and only harmonic biasing modulation with a single frequency $f_{b}$ is applicable; a ramp modulation signal containing higher frequency components, however, would be distorted by an uneven PEM response. Therefore, the solutions such as the dual ramp modulation [28] may ensure compensation within only a small region of phase shift, until the lowest PEM resonance frequency markedly affects the highest components of the ramp signal.

### 2.2. Closed-Loop Fully Harmonic Modulation

The novel solution to the closed-loop I-FOG utilizing the PEM as proposed herein is based on digital signal processing. In digital closed-loop I-FOGs, square-wave biasing modulation and synchronous sampling of the output with a period τ are typically used. By calculating the difference between odd and even samples, an open-loop signal is obtained, which then serves as the error signal for closed-loop compensation. In our case, an all-fiber piezoelectric modulator is employed, meaning that harmonic biasing modulation must be applied instead. Considering the output signal is sampled at the time instants corresponding to the peaks of the harmonic biasing modulation signal, both approaches lead to an identical digital signal. Compared to previous analog solutions, digital signal processing brings simple demodulation, zero electronic bias drift, and easy further processing.

**Figure 2:**
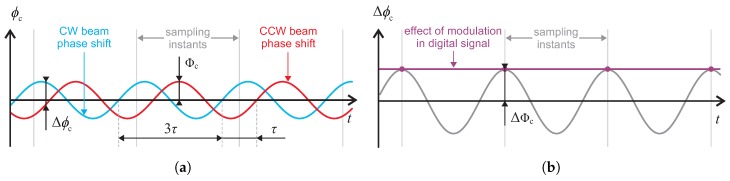
The proposed harmonic compensation modulation: the phase shift introduced to the CW and CCW beams (**a**), and their resulting phase difference (**b**).

As the open-loop signal is now discrete, the Sagnac effect does not have to be compensated continually all the time, unlike the ramp modulation scheme. Instead, the compensation can be ensured only at moments of sampling. The requirements on the modulation signal to compensate the Sagnac phase shift (further referred to as compensation modulation) can thus be reduced significantly. To compensate the Sagnac phase shift in the discrete open-loop signal, it is only necessary to achieve the desired phase difference at the sampling moments. In the present article, we attempt to demonstrate that this condition is satisfiable simply via another harmonic modulation signal, synchronous with the sampling process. In standard digital approaches to open and closed-loop I-FOGs, the intensity signal is usually sampled at the frequency of $f_{s}=2f_{b}=1/\tau$ to obtain alternating samples in positive and negative half-periods of the biasing modulation. In such a scenario, the harmonic compensation signal would have to null the Sagnac phase shift at every sampling instant; thus, it would need to have a period τ. This procedure, however, does not bring the required effect because the phase shifts introduced to the CW and CCW beams are also shifted by the τ; such a modulation would then have no impact, due to the CW and CCW beam phase shifts canceling. To eliminate this problem, we can use a different sampling frequency, $f_{s}^{\prime }$; we have $f_{s}^{\prime }=f_{s}/k$, where *k* represents any odd number greater than 1. The requirement for alternating the samples taken in positive and negative half-periods of the biasing modulation is thus retained, and the period between instants when the compensation needs to be ensured is extended to kτ. The nearest possible *k* is 3, and we thus choose the sampling and compensation modulation frequency to be $f_{s}^{\prime }=f_{c}=1/\left(3\tau \right)=\frac{2}{3}f_{b}$. The effect of the modulation on the phase of the beams in the CW and CCW directions is shown in Figure 2a, and the corresponding difference, $\Delta \phi_{c}$, between the CW and CCW phase shifts is displayed in Figure 2b. It can be seen that this difference introduces a steady phase shift at the sampling moments. By adjusting the compensation modulation amplitude, $\Phi_{c}$, we can directly compensate the nonreciprocal Sagnac phase shift at such moments. Since the sampling period is now 3τ, successive samples are acquired alternately in the positive and negative half-periods of the biasing modulation signal with frequency $f_{b}=1/\left(2\tau \right)$; in the process, two half-periods of the biasing modulation are always skipped. This allows us to obtain the open-loop signal in a normal manner, i.e., by calculating the difference between odd and even samples. Neglecting the fact that the sample rate decreases three-times compared to common open-loop operation, we can derive from Figure 2b that the closed-loop output signal ($\Delta \phi_{c}$) in the digital form is identical with the output signal obtained via the continuous ramp modulation shown in Figure 1.

This can be also proved mathematically. Considering the superposition of the Sagnac phase shift $\Delta \phi_{s}$, biasing modulation $\Delta \phi_{b}\left(t\right)$, and compensation modulation $\Delta \phi_{c}\left(t\right)$, the output signal after interference can be expressed as

(3) $$I\left(t\right)=I_{0}\left\{1+\cos\left[\Delta \phi_{s}+\Delta \phi_{b}\left(t\right)+\Delta \phi_{c}\left(t\right)\right]\right\},$$

where $I_{0}$ stands for the mean light intensity, or intensity without interference. Furthermore, the biasing modulation $\Delta \phi_{b}\left(t\right)$ and the compensation modulation $\Delta \phi_{c}\left(t\right)$ are expressed respectively as

(4) $$\Delta \phi_{b}\left(t\right)=\Delta \Phi_{b}\sin\left(\omega_{b}t\right),$$

(5) $$\Delta \phi_{c}\left(t\right)=\Delta \Phi_{c}\sin\left(\frac{2}{3}\omega_{b}t+\varphi \right),$$

where $\omega_{b}=2\pi f_{b}$, whereas φ is the phase delay of the compensation modulation. Note that both $\Delta \phi_{b}\left(t\right)$ and $\Delta \phi_{c}\left(t\right)$ are given as $\Delta \phi_{*}\left(t\right)=\phi_{*}\left(t\right)-\phi_{*}\left(t-\tau \right)$ because the phases of the CW and CCW beams are modulated with a delay equal to τ. As a consequence, $\Delta \Phi_{b}$ equals the double of the modulation amplitude since it operates at the proper frequency $f_{b}=1/\left(2\tau \right)$. The output signal given by Equation (3) is sampled with the period 3τ at instants corresponding to every third minima and maxima of the biasing modulation $\Delta \phi_{b}\left(t\right)$. Similarly to the common biasing modulation scheme, where the sampling period is τ, the samples that correspond to the minima and maxima instants alternate, albeit with a rate three times slower. The sampling instants exhibit the initial time shift τ/2 with respect to the biasing modulation, whose initial phase is zero. Thus, using substitution t→(3kτ−τ/2) to represent the above-mentioned sampling instants and utilizing Equations (4) and (5) and $\omega_{b}=\pi /\tau$, we obtain the discrete open-loop output as

(6) $$I\left(k\right)=I_{0}\left\{1+\cos\left[\Delta \phi_{s}+\Delta \Phi_{b}\sin\left(3k\pi -\frac{\pi }{2}\right)+\Delta \Phi_{c}\sin\left(2k\pi -\frac{\pi }{3}+\varphi \right)\right]\right\}.$$

By choosing the compensation modulation phase shift to be φ=−π/6, we make the total compensation modulation phase equal to $2k\pi -\frac{\pi }{2}$, and thus $\Delta \phi_{c}\left(k\right)=-\Delta \Phi_{c}$. As a result, the compensation modulation depth $\Delta \Phi_{c}$ can directly cancel out the Sagnac phase shift $\Delta \phi_{s}$ in the discrete output signal to produce the following output intensity at the sampling moments:

(7) $$I\left(k\right)=I_{0}\left\{1+\cos\left[\Delta \phi_{s}-\Delta \Phi_{c}+\Delta \Phi_{b}\sin\left(3k\pi -\frac{\pi }{2}\right)\right]\right\}.$$

Equation (7) can be rewritten as

(8) $$I\left(k\right)=I_{0}\left[1+A_{0}\left(\Delta \Phi_{b}\right)\cos\left(\Delta \phi_{s}-\Delta \Phi_{c}\right)-{\left(-1\right)}^{k}A_{1}\left(\Delta \Phi_{b}\right)\sin\left(\Delta \phi_{s}-\Delta \Phi_{c}\right)\right],$$

where $A_{0}\left(\Delta \Phi_{b}\right)$ and $A_{1}\left(\Delta \Phi_{b}\right)$ are the coefficients expressed as

(9) $$A_{0}\left(\Delta \Phi_{b}\right)=\cos\left(\Delta \Phi_{b}\right),$$

(10) $$A_{1}\left(\Delta \Phi_{b}\right)=\sin\left(\Delta \Phi_{b}\right).$$

By differentiating between the odd and even samples of I(k) in Equation (8), we suppress the direct component (the left and middle terms in the square brackets) to leave only the alternating term (the right one in the square brackets), which is multiplied by ${\left(-1\right)}^{k}$. We then obtain the desired open-loop signal

(11) $$S_{OL}=2I_{0}A_{1}\left(\Delta \Phi_{b}\right)\cdot \sin\left(\Delta \phi_{s}-\Delta \Phi_{c}\right).$$

As Equation (11) denotes, the Sagnac phase shift $\Delta \phi_{s}$ can be simply compensated by controlling the compensation modulation amplitude $\Delta \Phi_{c}$.

This modulation scheme has some notable advantages when used with a piezoelectric modulator. Instead of utilizing a ramp modulation signal with a constant amplitude and varying frequency, we employ a harmonic fixed-frequency signal with a variable amplitude. By adding this signal to the biasing modulation one, having frequency $f_{b}$, we obtain the resulting modulation signal. Since the signal contains only two harmonic components, we can make it fully compatible with the common PEM. However, care has to be taken to avoid the interference of the modulation and the PEM resonant frequencies, including their possible changes due to temperature drift. The resonant frequencies of the PEM are given by its dimensions and the applied piezoceramic material; both of these have to be chosen in accordance with the decisive parameter, namely, the length of the optical fiber and the corresponding transit time τ, which then determines the proper frequency $f_{b}$ and its 2/3-multiple.

Since the compensation effect is proportional to the amplitude of $\Delta \phi_{c}\left(t\right)$, the linearity of a closed-loop I-FOG depends on short samples’ acquisition and the linearity of the voltage-induced expansion of a piezoelectric modulator. The modulation sensitivity can be improved by increasing the number of the fiber wraps on the modulator; thus, the solution can provide linear compensation in a wide range of angular velocities.

### 2.3. Parasitic Effects of the Proposed Method

In a closed-loop sensor, the sensing quality is given by the quality of the feedback. Any distortion in the loop-back chain, caused primarily by the PEM and its driving circuits, may lead to the non-zero linearity error of the closed-loop I-FOG’s output characteristics. To conclude this section, we evaluate possible sources of distortion and propose an estimation of their effects on the total linearity error.

The first, well-known problem of piezoelectric actuators is hysteresis, which leads to not only different levels of compensation in the ascending and descending portions of the compensation signal but also nonlinear behavior of the PEM when high voltage is applied [22]. Another undesired effect is potential asymmetry between the PEM’s response to positive and negative voltages: whereas the expansion of the piezoceramic tube forces direct extension of the fiber, the contraction may be influenced by the elasticity of its attachment and the fiber tension preload. Both these effects cause the magnitude of the phase shift $\phi_{c}$ in the CW and CCW directions to be unequal at the sampling moments, resulting in closed-loop I-FOG output linearity distortion. Moreover, if the parasitic effects act simultaneously, the inequality appears also between distortions for positive and negative angular velocities.

Finally, we need to consider the limitations given by the PEM analog driving circuits, such as the saturation of the output amplifiers and the finite slew rate at higher compensation amplitudes $\Phi_{c}$. This saturation effect may manifests itself at higher angular velocities as a increase of the linearity error.

## 3. Experimental Setup

### 3.1. Optical Design

**Figure 3:**
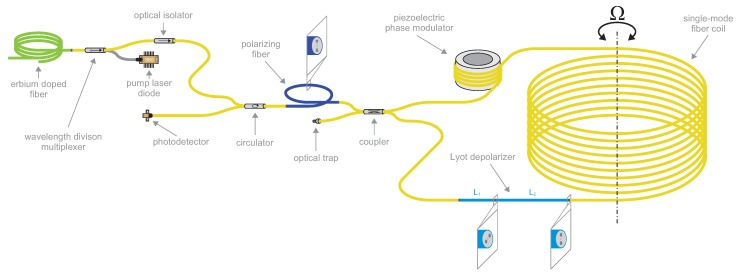
The optical architecture of the single-mode I-FOG setup for the PEM-based closed-loop method testing.

To test the proposed closed-loop method, we use a common I-FOG architecture with SM fiber [29]; a scheme of the tested setup is shown in Figure 3. The sensing coil comprises 770 m of SMF-28 fiber wound to the diameter of 160 mm; the fiber exhibits the total loss of 0.15 dB. We use quadrupolar winding to suppress the thermal transience effect.

**Figure 4:**
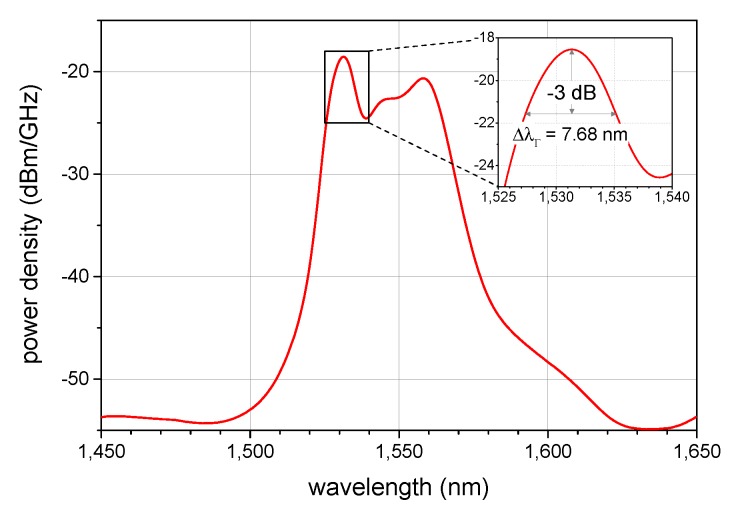
The output spectrum of the broadband superfluorescent fiber source utilized in the I-FOG setup (measured before the isolator).

As an I-FOG generally requires a broadband source, we employ a superfluorescent fiber source (SFS) consisting of a pump laser diode, erbium-doped fiber, and a wavelength division multiplexer. Compared to superluminescent diodes (SLD), which are commonly used in I-FOGs, the SFS produces naturally unpolarized light suitable for the SM fiber architecture. We employ a power-stabilized pump laser diode JDSU S26 7402-100 (Milpitas, CA, USA) with the central wavelength of 975 nm and maximal power of 100 mW. The active fiber is Fibercore M12-980-125 (Southampton, UK) with ≈12 dB/m absorption, calculated to have the length of 10 m for the maximum efficiency. The output broadband power reaches 36 mW, a value approaching the theoretical limit of the single-pass backward-output configuration, which is described in more detail within [30]. The output spectrum of the SFS source is shown in Figure 4. An optical isolator is used after the SFS to block potential radiation coming from the I-FOG.

The polarization state in the SM fiber may change randomly, causing a nonreciprocal phase difference between the CW and the CCW directions. To overcome this problem, we randomize the polarization state by employing a fiber Lyot depolarizer [11,13]. As a result, the nonreciprocal phase delay is distributed uniformly to all polarization states, and the reciprocity is ensured even with an SM fiber coil; a wider discussion of the problem is proposed in [31]. The depolarization effect exploits the low time coherence of the SFS. From the measured spectrum in Figure 4, we obtain the spectral width of $\Delta \lambda_{\Gamma }=7.68$ nm. The depolarizer comprises two segments of a Thorlabs PM1550-XP panda PM fiber (Newton, NJ, USA) with the beat length of Λ=5 mm (at 1550 nm); the segments exhibit the length ratio of $L_{1}:L_{2}=1:2$ and a turn of 45${}^{\circ }$ between their principal axes. To achieve effective depolarization, the shorter segment must be greater than or equal to the depolarizing length $L_{dp}$, expressed as

(12) $$L_{dp}=L_{dc}\cdot \Lambda /\lambda ,$$

where $L_{dc}$ denotes the decoherence length given by

(13) $$L_{dc}=\lambda^{2}/\Delta \lambda_{\Gamma }.$$

Assuming λ=1532 nm, we obtain $L_{dc}=306$ µm and $L_{dp}=0.997$ m to choose $L_{1}$ and $L_{2}$ equaling 1 and 2 m, respectively. The measured power loss introduced by the depolarizer was established to be about 1 dB (including the connectors).

To split and combine the input and output beams, we use an SM fiber circulator, and the beams in the CW and CCW paths are split and combined using a coupler; such a minimal configuration of the I-FOG helps us to preserve the reciprocity. A polarizer is placed in between the circulator and the coupler to compensate the imperfections of the Lyot depolarizer, as first demonstrated by [32]. The polarizer in our setup consists of a Thorlabs HB1550Z high-birefringent bow-tie polarizing fiber (Newton, NJ, USA) having the length of 4 m and spliced between the SM fibers, retaining the all-fiber setup.

To introduce the biasing and compensation phase modulations, we use a simple PEM in the form of a tube manufactured from hard piezoceramics (Noliac NCE40, Hradec Kralove, Czech Republic) and an ordinary SM fiber; the SM fiber is wound on the tube in several turns fixed with epoxy resin.

In order to estimate the total power loss, we first have to consider the filtering effect of the polarizing fiber, having 3 dB in each direction if the light coming from both directions is depolarized. Subsequently, the loss generated by the coupler will amount to 3 dB too. Then, some power loss may occur also in the fiber coil, including the PEM and the Lyot depolarizer. In our case, a standard telecom fiber was used for the PEM, resulting in the loss of 2.83 dB; however, the PEM’s loss may drop down to 1 dB if we use a bend-resistive fiber like that employed in the PEM by General Photonics (Chino, CA, USA).

### 3.2. Signal Processing and Closed-Loop Control

**Figure 5:**
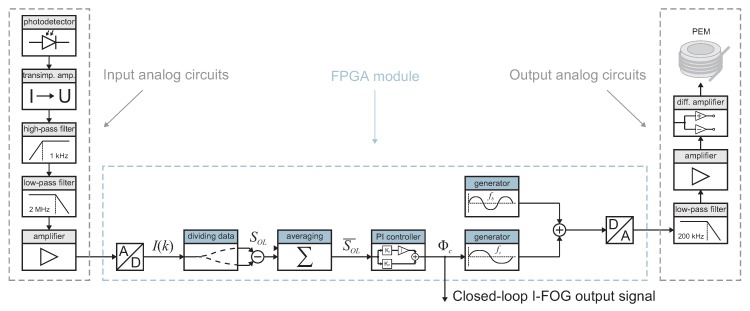
A diagram of the signal processing and the closed-loop control algorithm.

The signal processing and closed-loop control method is implemented into the applied field-programmable gate-array (FPGA) unit, which ensures high reliability and synchronizes the acquisition of the samples with the modulation signal generation. Our test setup utilizes a National Instruments PXI-7854R FPGA module (Austin, TX, USA) programmed in NI LabVIEW (version 2013, National Instruments, Austin, TX, USA). The diagram of the whole data processing chain is shown in Figure 5.

The fully digital processing ensured by the FPGA module is complemented with analog filtering and amplification circuits. The optical intensity signal, I(t), is converted to electric current using a Laser Components PDINP075FC83-W-0 (Olching, Germany) photodiode operating in the current mode to reduce the noise. This signal is converted to voltage by a transimpedance amplifier, which is followed by filters to suppress the DC component and high-frequency noise. The filtered signal is then matched to the A/D converter input range by another amplifier.

**Figure 6:**
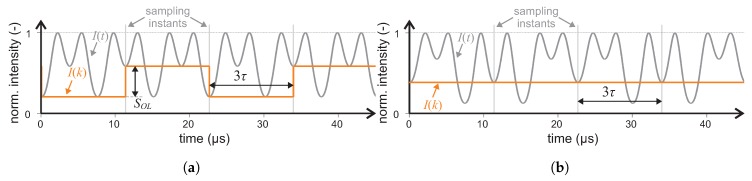
The time diagrams of the photodetected signals in the original I(t) and discrete I(k) forms, and the sampling instants for the uncompensated (**a**) and compensated (**b**) Sagnac phase shift. In (**a**), the sample difference corresponds to the error signal, whereas in (**b**) the zero error signal is detected.

Note that the anti-aliasing filter is omitted since the output is sampled by the A/D converter only at particular instants, as stated before. The sampling frequency is derived from the length of the fiber coil and the corresponding light transit time. In our case, the transit time is τ=3.78 µs, so the relevant biasing and compensation frequencies are $f_{b}=1/\left(2\tau \right)=132$ kHz and $f_{c}=\frac{2}{3}f_{m}=88$ kHz. The process of sampling the analog signal I(t) to yield the digital data I(k) is detailed in Figure 6. The case where a nonzero Sagnac phase shift $\Delta \phi_{s}$ is not compensated to produce $S_{OL}\neq 0$ is displayed in Figure 6a and the case with closed-loop compensation, where the $\Delta \phi_{s}$ is compensated by the $\Delta \Phi_{c}$ to result in $S_{OL}=0$ is presented in Figure 6b.

The digital signal I(k) (see Equation (7)) is then processed by the FPGA, which constitutes the core part of the signal processing chain. The open-loop signal $S_{OL}$ is obtained by calculating the amplitude difference between the odd and even samples of the I(k), as described before. The rate of the $S_{OL}$ thus decreases from 88 kS/s to $f_{c}/2=44$ kS/s. This is also the maximum rate of the whole control loop, allowing the theoretical maximum frequency of the angular velocity change to be in the order of several kHz. This open-loop signal, which actually embodies an error signal, then may be fed directly to the controller having a proportional (P) and an integrating (I) component. However, since there is always some noise in the $S_{OL}$ signal, this noise passes through the controller, emerging also in the closed-loop output signal and, thus, the compensation phase shift. In the worst case scenario, this may destabilize the closed-loop system, and it is then suitable to employ some averaging of the open-loop signal before it passes to the controller. In our setup, optional averaging by the factor of 10 is applied. Such a step will slow the control loop down to 4.4 kS/s, also reducing the closed-loop I-FOG bandwidth; conversely, it will save the FPGA resources and provide the output data, which are already filtered. The controller computes the current amplitude $\Phi_{c}$ of the compensation signal $\phi_{c}\left(t\right)$ created by the signal generator in the FPGA. The second generator shapes the biasing modulation $\phi_{b}\left(t\right)$, whose amplitude is constant. Both modulation signals are summed together and fed into the D/A converter. The rate of the D/A converter equals 1 MS/s in our implementation, and the analog signal is thus routed through the low-pass filter to suppress the corresponding quantization noise. Eventually, the smoothed signal level is adjusted to the input PEM’s range by the differential-output voltage amplifier.

**Figure 7:**
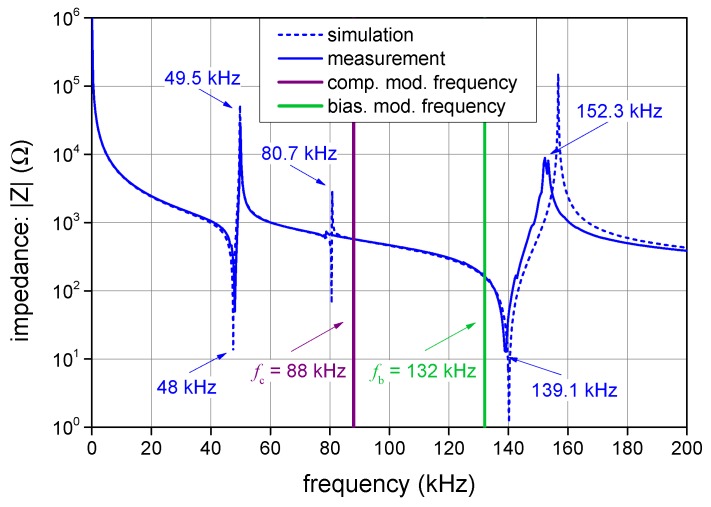
The frequency characteristics of the impedance of the employed PEM, with the biasing and compensation modulation frequencies marked in green and purple. The response was simulated using the finite element method and verified via experimental measurement.

Section 2.2 points out that the resonances of the PEM must avoid the modulation frequencies to ensure its stable operation. Assuming a PEM tube manufactured from the above-mentioned hard piezoceramics, we choose a tube exhibiting the outer diameter of 25.4 mm, height of 12.7 mm, and wall thickness of 3.15 mm having the lowest resonance frequencies at 50, 81, and 139 kHz, which satisfies the given condition, as shown in Figure 7.

## 4. Results and Discussion

**Figure 8:**
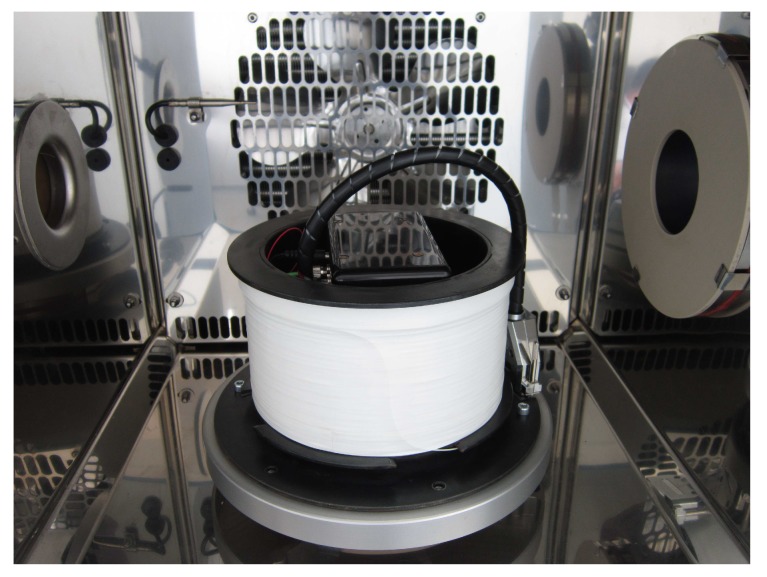
The experimental I-FOG setup tested on an RMS SDL1401 rotary table in a CTS T-65/50 temperature chamber.

To test the I-FOG utilizing the novel closed-loop method, we used an RMS SDL1401 precise calibration rotary table (RMS Regelungs- und Messtechnik GmbH & Co., KG, Hamburg, Germany) equipped with a CTS T-65/50 temperature chamber (CTS Clima Temperatur Systeme GmbH, Hechingen, Germany) to preserve stable conditions during the measurements. A photo of the described setup is presented in Figure 8. To demonstrate the advantages of the proposed compensation technique, we performed several tests of the I-FOG setup operating in both the open-loop and the closed-loop modes.

The closed-loop solution (Figure 5) was converted to the open-loop one by simply omitting the compensation modulation and leaving the averaged open-loop signal ${\bar{S}}_{OL}$ to be the I-FOG output. The sampling period was deliberately maintained at 3τ to facilitate straightforward comparison as its decrease to τ would not have exerted any substantial impact on the measured results. The open-loop setup thus performs in the same manner as the common open-loop I-FOGs with digital signal processing and constitutes a convenient reference.

### 4.1. Output Characteristics of the Open-Loop and Closed-Loop I-FOG

**Figure 9:**
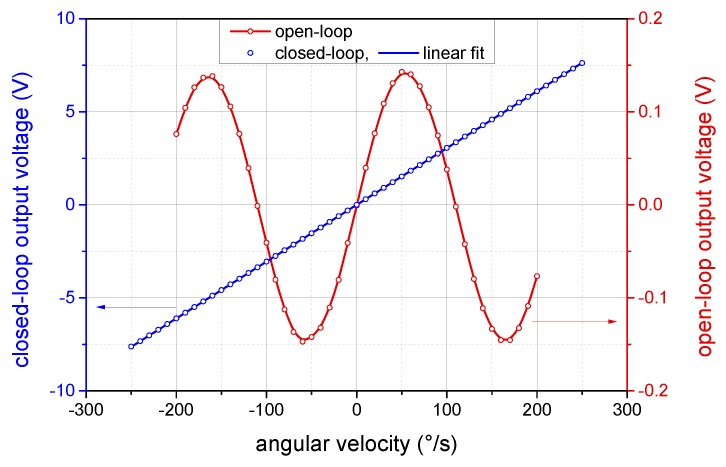
The output characteristics of the I-FOG measured during open-loop and closed-loop operation.

The measured dependence of the output voltage on the angular velocity for the standard open-loop and proposed closed-loop operation is shown in Figure 9. The measurements were performed at the temperature of 25 ${}^{\circ }$C. The open-loop output voltage is represented by the ${\bar{S}}_{OL}$ signal, whereas the closed-loop output corresponds to the compensation modulation amplitude $\Phi_{c}$. The maximum range of the angular velocities measured in closed-loop operation is thus limited by the output voltage range, which, in our case, was ±10 V. However, the effective range is further limited by the presence of stable biasing modulation; thus, the useful range is only about ±6.6 V. This allows us to compensate the angular velocities in the range of ±250${}^{\circ }$/s, as shown in Figure 9. Assuming λ=1532 nm and considering the coil and fiber dimensions mentioned in Section 3.1, the corresponding maximum Sagnac phase difference amounts to ±7.35 rad. Further extension of the closed-loop compensation range is possible through increasing the number of the fiber wraps on the modulator or via higher driving voltage.

### 4.2. Output Linearity

**Figure 10:**
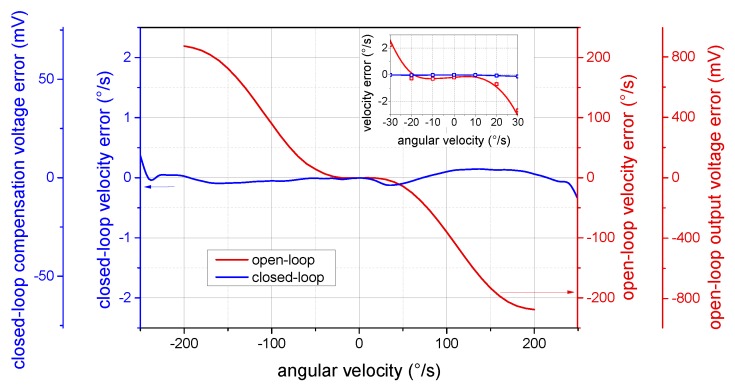
The deviation of the measured output characteristics of the open-loop and closed-loop I-FOG from their ideal linear approximations. The output voltage and the corresponding angular velocity errors are scaled by the vertical axes. A detailed view of the small angular velocities is shown in the inset.

**Figure 11:**
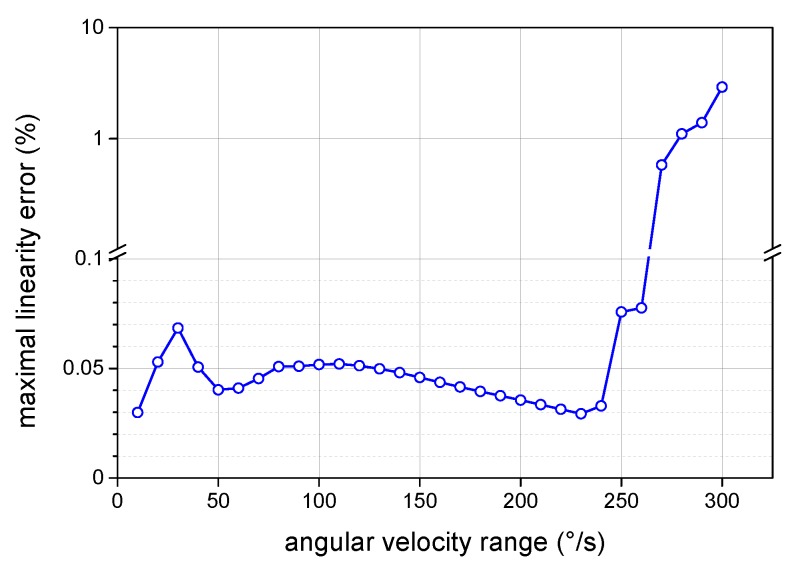
The dependence of the closed-loop I-FOG standard linearity error on the considered angular velocity range.

The linearized and unambiguous output response is considered as one of the benefits of closed-loop operation. In theory, the proposed method allows for achieving very good linearity assuming precise control of the modulation depth. Nevertheless, practically, the above-mentioned imperfections bring some residual nonlinearity, which must be taken into account for higher-grade applications. The deviation of the measured response from its linear fitting is shown in Figure 10. Whereas the sine-shaped open-loop characteristics deviate even for small angular velocities, the closed-loop response remains linear within the considered range of ±250${}^{\circ }$/s, with the maximum error of 0.37${}^{\circ }$/s at the range edges. The standard linearity error was calculated as

(14) $$\delta_{L}\left(\Omega_{range}\right)={\left\{\left|\frac{\Omega_{M}-\Omega_{L}}{\Omega_{max}-\Omega_{min}}\right|\right\}}_{\max}\cdot 100\;\left(\%\right),$$

where $\Omega_{M}$ is the measured angular velocity and $\Omega_{L}$ its linear approximation by the least square method within the given input angular velocity range $\Omega_{range}$. Furthermore, $\Omega_{max}$ and $\Omega_{min}$ are the upper and lower limits of $\Omega_{M}$ within the input angular velocity range $\Omega_{range}$. This dependence (Figure 11) exhibits an increase of the linearity error above ±260${}^{\circ }$/s, caused by reaching the maximal compensation voltage provided by the driving circuits of the tested I-FOG prototype. Within this range, the maximal linearity error is below 0.08% for any considered subrange. The residual nonlinearity observed in the closed-loop output response can be explained by the presence of parasitic effects, as discussed in Section 2.3.

### 4.3. Temperature Dependence

Although most fiber-optic sensors are temperature-dependent, the minimal I-FOG architecture is sufficiently resistant due to its reciprocity, independence of the fiber refractive index, and the advanced winding types suppressing the effect of temperature transience. Therefore, we will focus here especially on the impact of temperature upon the phase shift compensation, which plays a key role in the novel closed-loop method.

**Figure 12:**
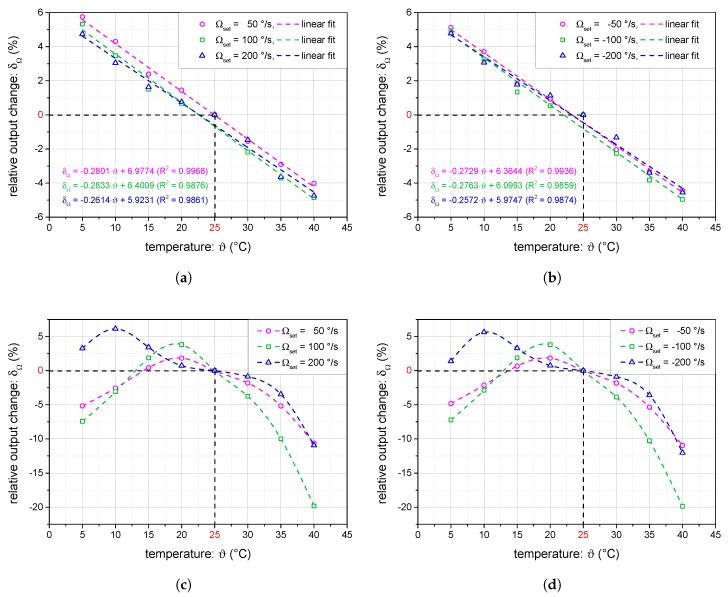
The measured temperature-dependent relative change of the I-FOG output signal for closed-loop (**a**,**b**) and open-loop (**c**,**d**) operation ($\vartheta_{ref}=25$ ${}^{\circ }$C).

The experiments were performed within the temperature range of 5–40 ${}^{\circ }$C. The measured angular velocity was referred to the value detected at the temperature of 25 ${}^{\circ }$C and expressed as a relative change. The results for the open-loop and the closed-loop operation modes are compared in Figure 12a–d. The waiting time at each temperature was over four hours to ensure precise temperature settling. In each of the temperatures, we acquired data at six different angular velocities (±50, ±100 and ±200${}^{\circ }$/s). At each angular velocity, the output data were averaged after more than one minute of datalogging to avoid the noise.

The results for the closed-loop operation in Figure 12a,b indicate that the temperature dependence does not vary significantly for different angular velocities. Furthermore, the dependence can be approximated linearly, with the slope being about −0.27%/${}^{\circ }$C. This change is in accordance with the temperature drift of the piezoelectric coefficients of the hard piezoceramic materials used to construct the PEM, as can be found in e.g., Ref. [27], providing a broader discussion of the piezoelectric materials temperature dependence. Note that the piezoelectric coefficients change with the opposite sign as they are indirectly proportional to the input voltage required for the desired phase shift compensation.

Compared to the closed-loop operation, the temperature dependence of the relative output in the open-loop I-FOG setup is highly nonlinear. Even though the compensation modulation is not present, the temperature dependence of the PEM’s piezoelectric coefficients will manifest itself through the biasing modulation. In the open-loop setup, the change of the biasing modulation depth $\Phi_{b}$ has a direct influence on the open-loop signal. Moreover, the relative change varies with the Sagnac phase shift, i.e., the angular velocity. Another drawback of open-loop operation consists of the dependence on the light source. The temperature can have an impact on the total power as well as the spectral shape of the broadband source; this may lead to more complex shapes of the temperature dependence, as shown in Figure 12c,d, where the effects are combined and thus become markedly more difficult to compensate.

### 4.4. Allan Deviation and Time Stability

**Figure 13:**
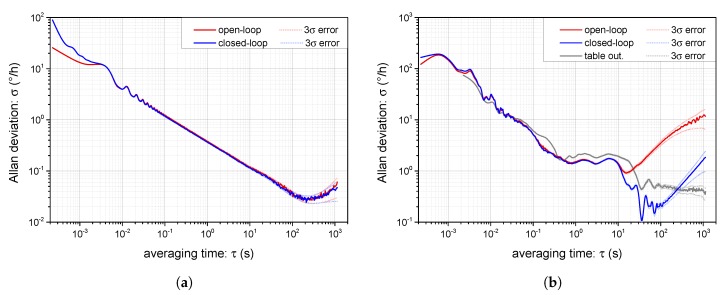
The measured Allan deviation for closed-loop and open-loop I-FOG operation at the zero table angular rate (**a**) and the angular rate of 10${}^{\circ }$/s with the direct table velocity output (**b**); the 3σ error boundaries are marked with the dotted lines.

To express the long-term stability of fiber-optic gyroscopes, the Allan variance or deviation is used as a standard parameter to average the output signal over an increasing period. The methodology of determining the Allan deviation for I-FOGs is described in detail within IEEE standards [33]. According to these, we measured the Allan deviation σ in the closed-loop and the open-loop I-FOG setups. The results for the zero table rotation (only the Earth’ orthogonal component is present) are shown in Figure 13a. Both of the curves coincide, as originally assumed; this proves that the proposed closed-loop control algorithm and compensation modulation do not introduce any additional noise, unlike the open-loop setup. The bias stability observed at the zero rate was better than 0.03${}^{\circ }$/h, and the angle random walk (ARW) was approximately 0.006${}^{\circ }/\sqrt{h}$. The results indicate that the method can be usefully applied in I-FOGs of intermediate and terrestrial navigation levels without impairing their inherent stability.

Note that, although only 16-bit A/D and D/A converters with ±10 V ranges are utilized, which causes the quantization level to equal 0.012${}^{\circ }$/s in closed-loop operation, the dithering by the white noise present in the closed-loop system and the high resolution in time given by the closed-loop algorithm reaching up to 44 kS/s (in our test setup, the rate was reduced to 4.4 kS/s, as stated above) enables us to obtain accuracy rates of down to 0.03${}^{\circ }$/h.

Whereas the open-loop and closed-loop signals for the zero rate coincide, at non-zero angular velocities, we observed a slow drift of the open-loop output signal. This effect is typically caused by instability of the SFS, even when the pump laser diode is power-stabilized. At the Earth’ rotation rate, the difference between consecutive samples is almost zero and is thus overcome by the white noise of the detector and analog signal pre-processing. However, in the case of a higher rotation rate, the white noise becomes overwhelmed by the dependence of the output on the input power, which affects the open-loop output correspondingly. In a closed-loop I-FOG, the problem can be avoided easily as the open-loop signal is maintained at zero by the feedback. To demonstrate this effect, we measured the Allan deviation with the table angular velocity set to 10${}^{\circ }$/s. The results are shown in Figure 13b, where, compared to Figure 13a, the Allan deviation is highly distorted by other sources of noise. These sources include, above all, the rotation table bearing and table motion controller, as is also obvious from the direct table velocity output, whose Allan deviation is included in Figure 13b. Comparing the Allan deviation of the open-loop and closed-loop I-FOG rotating at 10${}^{\circ }$/s, we can see in the open-loop operation mode the negative effect of source power fluctuation, a difficulty eliminated by the closed-loop setup. In our case, closed-loop operation attains a stability higher by about one order with fine angular velocity resolutions and at long averaging times.

Note that all the I-FOG data logged to calculate the Allan variance and deviation were sampled with the period of 3τ×20=226.8 µs, where the multiplication by 20 is given by computing the difference between consecutive samples and averaging process, as remarked in Section 3.2. In Figure 13a,b, we also show the estimated Allan deviation reliability for a 3σ error calculated according to [33].

### 4.5. Closed-Loop System Dynamic Performances

A potential drawback of any closed-loop sensing system consists of limited bandwidth, restricted by the speed of the control loop. In many cases, a compromise has to be found between the high bandwidth and the stability of the system. With the proposed compensation method, the maximum error signal rate corresponds to 1/(6τ)=44 kS/s; the rate is determined by the transit time τ given by the fiber length. As mentioned previously, we employ averaging by the factor of 10, and the actual control loop rate thus decreases to 4.4 kS/s in our test setup. Compared to open-loop operation, the proposed closed-loop method exhibits a fundamental bandwidth limitation given by the sampling frequency drop from $f_{s}=1/\tau$ to $f_{s}^{\prime }=1/\left(3\tau \right)$. Although we showed in Section 2 that the proposed harmonic closed-loop method is equivalent to the digital ramp modulation approach in discrete signals, the decrease of the fundamental sampling rate by the factor of 3 means that the maximum bandwidth of the closed-loop I-FOG is at least three times smaller than in the open-loop one.

**Figure 14:**
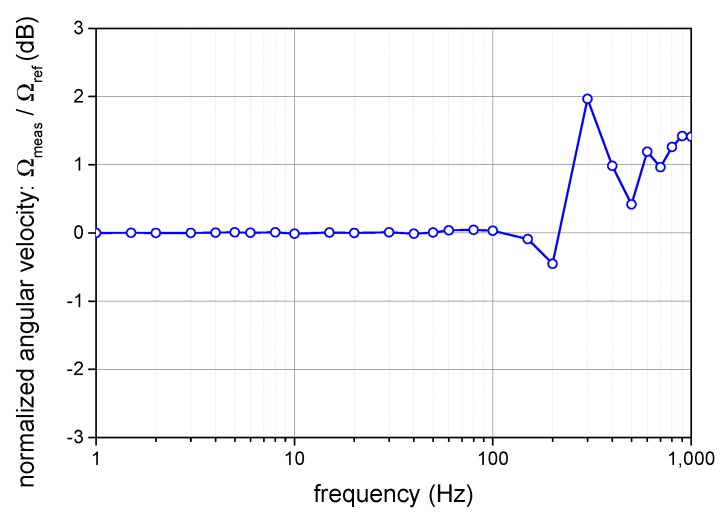
The frequency characteristics of the measured sine shape angular velocity at the calibration rotation table (the maximum closed-loop control rate was employed).

To verify the limits of the closed-loop I-FOG, we applied sine angular vibrations to the test setup and observed the output signal. As the reference, we utilized a Polytec OFV-505 laser vibrometer (Waldbronn, Germany) to sense the longitudinal vibrations of the reflective target placed on the outer side of the I-FOG. The frequency characteristics of the closed-loop I-FOG are displayed in Figure 14. The response remains flat up to 100 Hz, after which some irregularities begin to appear due to the total control loop time delay, the control dynamics and averaging. However, the 3dB-bandwidth is broader than 1 kHz, and without averaging and closed-loop signal delay, it could reach beyond several kHz to satisfy the demands of some high-grade applications.

### 4.6. Comparing the Proposed Harmonic Closed-Loop and the Standard Open-Loop Configurations

The novel closed-loop method was experimentally tested using the common all-fiber I-FOG architecture based on ordinary SM fiber and PEM. The setup was also characterized as convertible into a standard open-loop I-FOG with only minor changes. Such flexibility facilitated direct comparison of the proposed closed-loop method with harmonic signal compensation and the common approach to low-cost I-FOG utilizing SM fiber and PEM. Exploiting the experimental results, Table 1 summarizes the major parameters of the test I-FOG operating in the open-loop and closed-loop modes.

## 5. Conclusions

We presented a novel and simple method enabling closed-loop operation of the PEM-based I-FOG with an inexpensive all-fiber architecture. Compared to the common concept of the closed-loop I-FOG with sawtooth modulation, requiring a broadband electro-optic modulator with a polarizer and additional depolarizers (assuming depolarized light and SM fiber), we employ a harmonic modulation signal to compensate the Sagnac phase shift. Such a solution enables us to utilize a piezoelectric modulator and all-fiber components, which are otherwise reserved for simple open-loop I-FOGs. The compensation method consists of introducing another harmonic phase modulation to the biasing modulation, whose frequencies are related as defined by $f_{c}=\frac{2}{3}f_{b}$, and synchronous sampling of the output at moments of compensation. This technique is applicable to basically any open-loop I-FOG without needing optical architecture modification, and improves its performances by the closed-loop compensation.

The main advantages of closed-loop operation mode, as also shown in the article, include insensitivity to the source power fluctuations and also linearization and unambiguity of the angular velocity detection with stable accuracy within a wide range. Using our tested setup, we achieved a closed-loop compensation of the Sagnac phase shift within the range of ±250${}^{\circ }$/s. One significant benefits is also the improved temperature dependence, which became approximately linear and invariant with the angular velocity; thus, it can be easily compensated if the temperature of the piezoelectric modulator is measured at the same time. Compared to the highest-grade closed-loop I-FOGs utilizing an electro-optic modulator and polarizing components, the residual output nonlinearity is higher due to the aforementioned PEM parasitic effects. However, we believe that this residual nonlinearity could be considerably decreased by suitable post processing, e.g., by a neural network-based approach successfully adopted in open-loop I-FOG [34]. Furthermore, due to suppression of the temporal instability of the source, the bias stability was enhanced in the closed-loop setup by more than one order when the system was rotating.

Reducing the cost of the I-FOG technology is a practical problem attracting serious interest within the given domain. One of the commercially viable approaches is using ordinary single mode-fiber and an all-fiber architecture with a piezoelectric modulator. We therefore assume that adopting the advantages of the proposed closed-loop processing method could improve the performance of lower cost I-FOGs based on the above architecture, with the main target applications being in terrestrial navigation and diverse branches of industry where the manufacturing simplicity and high reliability are of high importance.

## Figures and Tables

**Table 1:** The parameters of the tested I-FOG prototype in the open-loop and closed-loop modes.

I-FOG Parameter	Open-Loop	Closed-Loop
bias stability	0.03${}^{\circ }$/h	0.03${}^{\circ }$/h
ARW	0.006${}^{\circ }/\sqrt{h}$	0.006${}^{\circ }/\sqrt{h}$
SFS stability (at 10${}^{\circ }$/s)	≈1${}^{\circ }$/h	no impact
linearity error	nonlinear	<0.08% (±250${}^{\circ }$/s)
temp. dependence (5–40 ${}^{\circ }$C)	sine	linearized

## Abbreviations

The following abbreviations are used in this manuscript:

ARW	angle random walk
FPGA	field-programmable gate-array
I-FOG	interferometric fiber-optic gyroscope
MEMS	microelectromechanical systems
PEM	piezoelectric modulator
PM	polarization-maintaining
RLG	ring laser gyroscope
SFS	superfluorescent fiber source
SLD	superluminescent diode
SM	single-mode
